# Mobile Carbapenemase Genes in *Pseudomonas aeruginosa*

**DOI:** 10.3389/fmicb.2021.614058

**Published:** 2021-02-18

**Authors:** Eun-Jeong Yoon, Seok Hoon Jeong

**Affiliations:** Department of Laboratory Medicine and Research Institute of Bacterial Resistance, Yonsei University College of Medicine, Seoul, South Korea

**Keywords:** carbapenem resistance, carbapenemase, molecular epidemiology, *Pseudomonas aeruginosa*, mobile genetic elements, genomic islands

## Abstract

Carbapenem-resistant *Pseudomonas aeruginosa* is one of the major concerns in clinical settings impelling a great challenge to antimicrobial therapy for patients with infections caused by the pathogen. While membrane permeability, together with derepression of the intrinsic beta-lactamase gene, is the global prevailing mechanism of carbapenem resistance in *P. aeruginosa*, the acquired genes for carbapenemases need special attention because horizontal gene transfer through mobile genetic elements, such as integrons, transposons, plasmids, and integrative and conjugative elements, could accelerate the dissemination of the carbapenem-resistant *P. aeruginosa*. This review aimed to illustrate epidemiologically the carbapenem resistance in *P. aeruginosa*, including the resistance rates worldwide and the carbapenemase-encoding genes along with the mobile genetic elements responsible for the horizontal dissemination of the drug resistance determinants. Moreover, the modular mobile elements including the carbapenemase-encoding gene, also known as the *P. aeruginosa* resistance islands, are scrutinized mostly for their structures.

## Introduction

*Pseudomonas aeruginosa* is a non-fermentative and aerobic Gram-negative bacillus that is one of the leading causes of severe health care-associated infections targeting immunocompromised patients (Rice, 2008). The bacterial species is an opportunistic pathogen not only for humans but also for plants and animals. *P. aeruginosa* is metabolically versatile, and it has an enormous ability for adaptation to different conditions with genome plasticity (Shen et al., 2006). There are diverse opinions whether the pangenome of *P. aeruginosa* is still open or closed to acquire foreign genes (Klockgether et al., 2011; Mosquera-Rendon et al., 2016). The accessory genome is often composed of genes involved in virulence to human hosts and antimicrobial resistance, resulting in a high risk of mortality and a high rate of multidrug resistance (Moradali et al., 2017). *P. aeruginosa* has a median genome size value of 6.7 Mbp, a median number of 6,016 coding sequences, and 66.1% GC on average (NCBI, 2020).

Although beta-lactams are one of the most commonly used antimicrobial drug classes for *P. aeruginosa* infection, antipseudomonal beta-lactam drugs are limited because of the species’ intrinsic resistance due to the interplay of chromosomal beta-lactamases (Livermore and Yang, 1987), a low outer membrane permeability (Angus et al., 1982), and the constitutive expression of efflux pump systems (Li et al., 1995). The beta-lactam regimens for *P. aeruginosa* infection include antipseudomonal penicillins in combination with a beta-lactamase inhibitor, i.e., piperacillin–tazobactam and ticarcillin–clavulanic acid; antipseudomonal cephalosporins alone or in combination with beta-lactamase inhibitors, i.e., ceftazidime (with avibactam), ceftolozane (with tazobactam), cefoperazone (with sulbactam), and cefepime (Slack, 1981); and carbapenems, i.e., imipenem and meropenem. Among those, carbapenems are the preferred choice against multidrug-resistant *P. aeruginosa*. In recent years, the rate of carbapenem resistance in *P. aeruginosa* has increased worldwide and has become of great concern since it significantly restricts the therapeutic options for patients (El Solh and Alhajhusain, 2009). Carbapenem resistance in *P. aeruginosa* is caused by chromosomal substitutions resulting in membrane permeability alterations through porin loss and efflux pump overexpression, together with intrinsic beta-lactamase derepression, and the acquisition of the genes for carbapenemases (Livermore, 1992; Masuda et al., 2000; Lister et al., 2009).

In this review, we summarized carbapenem resistance in *P. aeruginosa*. The first half of this review outlines the worldwide epidemiology and the second half discusses the mechanisms of carbapenem resistance and the mobile genetic elements responsible for the horizontal dissemination of resistance determinants. All of the information in the review was collected and analyzed from the National Database of Antibiotic Resistant Organisms^1^ using the Reference Gene Catalog ver. 2020-09-22.2^2^ for the carbapenemases and the Genome Database (last updated on 22 September 2020)^3^ for the complete *P. aeruginosa* genomes.

## Carbapenems for Antipseudomonal Treatment and Resistance in *P. aeruginosa*

### Carbapenems for the Treatment of Patients With *P. aeruginosa* Infection

**FIGURE 1:**
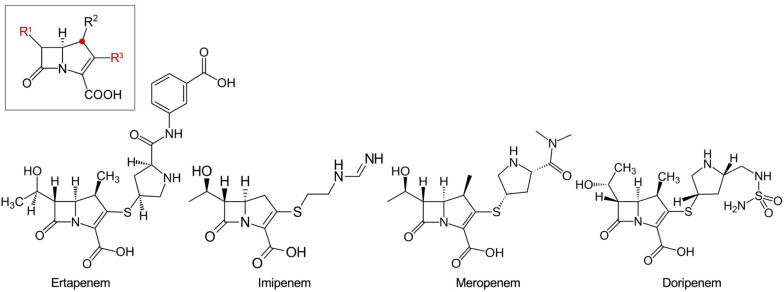
Carbapenem drugs. The backbone of carbapenems is in a *box*. The C-1 position replaced from the sulfur atom in the penicillin backbone is indicated with a *red dot*. The important R1 and R3 positions are indicated with *red letters*. Ertapenem with a bulky R3 residue, which does not have enough affinity to be active against *Pseudomonas aeruginosa*, is presented with the other three carbapenems having antipseudomonal activity.

Beta-lactams act by binding to and inactivating the penicillin-binding proteins (PBPs), which have an essential role for the completion of peptidoglycan biosynthesis through their dual activity as a transglycosylase and transpeptidase. Among the beta-lactams, carbapenems are the most effective against Gram-positive and Gram-negative bacteria, presenting a broad spectrum of antibacterial activity. Replacing the sulfur atom at the C-1 position of the penicillin backbone by a carbon atom (the red dot of the carbapenem backbone in the box in Figure 1) allows exceptional stability against most enzymes inactivating beta-lactams (Papp-Wallace et al., 2011).

**FIGURE 2:**
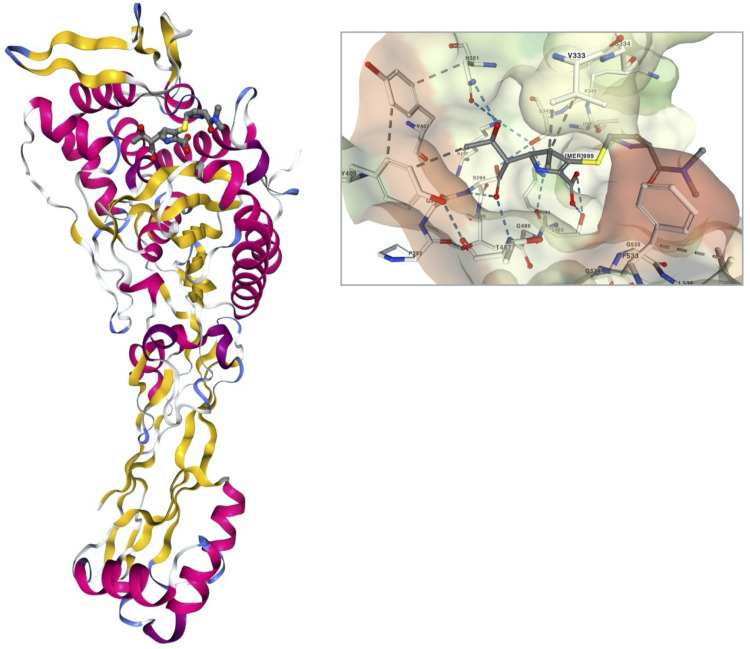
A stereoview of PBP3 of *Pseudomonas aeruginosa* complexed with meropenem (PDB ID, 3PBR) and the interaction of the meropenem in the ligand pocket of PBP3 (Han et al., 2010). The structure of PBP3 is colored by secondary structure, and the meropenem is in a ball-and-stick presentation. The molecular surface of PBP3 in the binding pocket is presented with the interacting amino acid residue complex with meropenem in a ball-and-stick presentation.

The early carbapenem ertapenem has a bulky R3 moiety, resulting in a weak affinity to the PBP3 of *P. aeruginosa*, and consequently, the drug has little activity against *P. aeruginosa* (Figure 1). Thus, the late carbapenems imipenem and meropenem are actively used for the treatment of infections (Luyt et al., 2014). Among the three antipseudomonal carbapenems, imipenem has a distinctive stereographic structure and the lowest minimum inhibitory concentrations (MICs) for wild *P. aeruginosa* strains (Figure 1). Meropenem presents good affinity to the active site of PBP3 (Figure 2). The recently developed combinations of beta-lactam/beta-lactamase inhibitor, such as aztreonam–avibactam, meropenem–vaborbactam, and imipenem–relebactam, have a limited efficacy against the metallo-beta-lactamase (MBL)-producing carbapenem-resistant *P. aeruginosa* (Karlowsky et al., 2017, 2020; Lob et al., 2020). However, the siderophore cephalosporin cefiderocol (Delgado-Valverde et al., 2020) is effective against all the carbapenemase-producing *P. aeruginosa*, including the MBL producers.

### Carbapenem Resistance in *P. aeruginosa*

**FIGURE 3:**
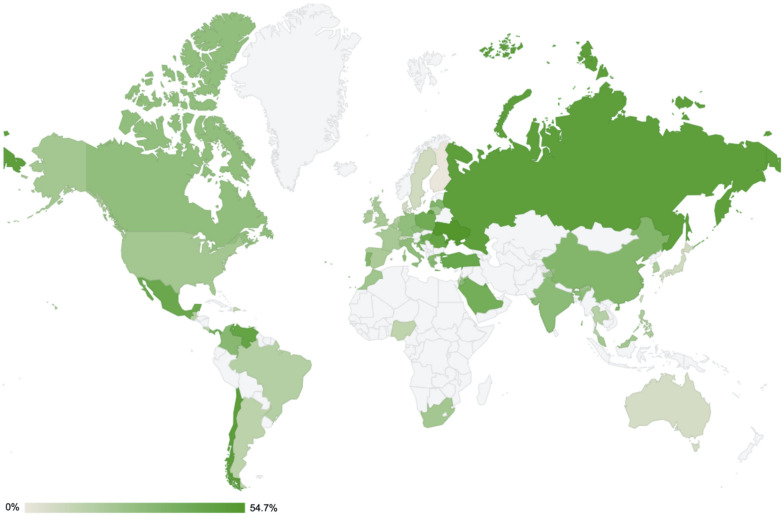
Rates of imipenem resistance in *Pseudomonas aeruginosa* worldwide in 2018. All the data were extracted from the Antimicrobial Testing Leadership and Surveillance run by Pfizer (last updated on October 30, 2019) (Pfizer, 2020), except for the data from South Korea (unpublished data). The actual resistance rates are indicated per continent as follows: Africa [three participating countries: Morocco (*N* = 79, 22.8%), Nigeria (*N* = 38, 13.2%), South Africa (*N* = 98, 21.4%)]; Asia [10 participating countries: China (*N* = 386, 33.2%), Hong Kong (*N* = 25, 24%), India (*N* = 125, 29.6%), Japan (*N* = 75, 8%), Malaysia (*N* = 55, 21.8%), Philippines (*N* = 74, 18.9%), Singapore (*N* = 25, 16%), South Korea (*N* = 127, 18.1%), Taiwan (*N* = 99, 9.1%), and Thailand (*N* = 75, 16%)]; Europe [24 participating countries: Belgium (*N* = 150, 21.3%), Croatia (*N* = 78, 35.9%), Czech Republic (*N* = 102, 29.4%), Denmark (*N* = 25, 8%), Finland (*N* = 19, 0%), France (*N* = 248, 20.2%), Germany (*N* = 248, 27.4%), Greece (*N* = 75, 30.7%), Hungary (*N* = 100, 42%), Ireland (*N* = 64, 18.8%), Italy (*N* = 242, 28.5%), Latvia (*N* = 24, 33.3%), Lithuania (*N* = 50, 22%), Poland (*N* = 101, 39.6%), Portugal (*N* = 99, 32.3%), Romania (*N* = 99, 45.5%), Russia (*N* = 194, 48.5%), Netherlands (*N* = 40, 17.5%), Spain (*N* = 273, 19.4%), Sweden (*N* = 25, 8%), Switzerland (*N* = 50, 10%), Turkey (*N* = 60, 43.3%), Ukraine (*N* = 53, 54.7%), and United Kingdom (*N* = 158, 19.6%)]; Mid and South America [10 participating countries: Brazil (*N* = 116, 16.4%), Chile (*N* = 75, 49.3%), Colombia (*N* = 124, 30.7%), Costa Rica (*N* = 22, 13.6%), Dominican Republic (*N* = 24, 12.5%), Guatemala (*N* = 55, 16.4%), Mexico (*N* = 146, 42.5%), Panama (*N* = 32, 37.5%), and Venezuela (*N* = 64, 45.3%)]; North America [two participating countries: Canada (*N* = 197, 27.4%) and the United States (*N* = 588, 21.4%),]; Middle East [four participating countries: Israel (*N* = 100, 12%), Jordan (*N* = 24, 29.2%), Kuwait (*N* = 76, 44.7%), and Saudi Arabia (*N* = 26, 38.5%)]; and Oceania [a single participating country: Australia (*N* = 99, 7.1%)].

The rate of carbapenem resistance in *P. aeruginosa* varies worldwide (Figure 3). According to the Antimicrobial Testing Leadership and Surveillance program by Pfizer in 2018 (last updated on September 14, 2020) (Pfizer, 2020), the rate of resistant clinical strains (-R) of *P. aeruginosa* by continent was the lowest in Oceania (imipenem-R in 7.1% and meropenem-R in 5.1% of 99 isolates from Australia), and the highest was in the Middle East (imipenem-R in 27.9% and meropenem-R in 19.5% of 226 isolates from four participating countries). In descending order, the median resistance rates were 30.7% in South America (the lowest imipenem-R and meropenem-R both in 12.5% of 24 isolates from the Dominican Republic and the highest imipenem-R in 49.3% and meropenem-R in 75.3% in 75 isolates from Chile, among nine participating countries), 28.0% in Europe (the lowest 0.0% of 19 isolates from Finland and the highest imipenem-R in 48.5% and meropenem-R in 44.8% of 194 isolates from Russia, among 24 participating countries), 24.4% in North America (imipenem-R in 21.4% and meropenem-R in 18.3% of 197 isolates from Canada and imipenem-R in 27.4% and meropenem-R in 15.5% of 588 isolates from the United States), 22.8% in Africa (the lowest imipenem-R in 13.2% and meropenem-R in 15.8% of 38 isolates from Nigeria and the highest imipenem-R in 21.4% and meropenem-R in 19.4% of 98 isolates from South Africa, among three participating countries), and 18.1% in Asia (the lowest imipenem-R and meropenem-R both in 8.0% of 75 isolates from Japan and the highest imipenem-R in 33.2% and meropenem-R in 25.1% of 386 isolates from China, among 10 participating countries).

### Epidemic High-Risk *P. aeruginosa* Clones

The current *P. aeruginosa* high-risk clones, which meet both requirements of global dominance and association with the multidrug-resistant phenotype, include 10 *P. aeruginosa* lineages belonging to ST111, ST175, ST233, ST235, ST244, ST277, ST298 (CC445), ST308, ST357, and ST654 (Del Barrio-Tofino et al., 2020). The multidrug-resistant *P. aeruginosa* ST111, ST175, and ST235 have been identified to carry genomic islands (Roy Chowdhury et al., 2017). While all the 10 high-risk clones are relevant to MBL production, ST235 and ST111 are by far the most worrisome carbapenemase producers, associated not only with class B but also with class A and D carbapenemases. The widespread *P. aeruginosa* ST235 clone is often associated with poor clinical outcomes due to its multidrug resistance and virulence factors, representatively the cytotoxin ExoU causing necrotic cell death (Sato et al., 2003; Roy Chowdhury et al., 2016; Yoon et al., 2019). The second dominant *P. aeruginosa* clone is ST111, which has been identified in all six continents except Oceania (Del Barrio-Tofino et al., 2020).

## Mechanisms of Resistance to Carbapenems in *P. aeruginosa*

### Chromosomal Mutation-Derived Carbapenem Resistance

*Pseudomonas aeruginosa* can acquire resistance to carbapenems by chromosomal mutations (Lister et al., 2009). Loss of the outer membrane protein OprD, which is a channel for imipenem penetration (Margaret et al., 1989), is associated with a reduced susceptibility to carbapenems, mostly imipenem (Farra et al., 2008). Early reports have underlined OprD deficiency as the predominant mechanism of carbapenem resistance in *P. aeruginosa* (Margaret et al., 1989; Kohler et al., 1999). The overexpression of efflux pump systems, such as MexAB-OprM, by mutation at the regulatory region contributes directly to the resistance to meropenem (Kohler et al., 1999; Masuda et al., 2000) and mutational derepression of the chromosomal cephalosporinase AmpC, especially the extended-spectrum cephalosporinases (Rodriguez-Martinez et al., 2009a), and plays a part in carbapenem resistance (Quale et al., 2006; Rodriguez-Martinez et al., 2009b). The combination of porin loss, efflux pump overexpression, and chromosomal cephalosporinase derepression is able to confer high-level resistance to carbapenems, and *P. aeruginosa* could have elevated imipenem and meropenem MICs up to 256 and 128 mg/L, respectively (Chalhoub et al., 2016).

### Enzymatic Mechanisms of Carbapenem Resistance

Before 1990, the only known mechanism of carbapenem resistance was mutations occurring in the chromosome. Following the first identification of an MBL-producing *P. aeruginosa* clinical strain (Watanabe et al., 1991; Minami et al., 1996), a retrospective screening of *P. aeruginosa* identified the *bla*_IMP–1_ gene in 1992 in Japan (Senda et al., 1996a). Subsequent outbreaks due to the transferable drug resistance conferred by the gene were reported (Senda et al., 1996b). The *bla*_VIM–1_ gene encoding the Verona integron-encoded MBL (VIM) subtype 1 in *P. aeruginosa* clinical strain was identified in 1997 in Italy in a *P. aeruginosa* clinical isolate (Lauretti et al., 1999). And the carbapenem-resistant *P. aeruginosa* spread rapidly through the contribution of mobile genetic elements and high-risk clones. Thus far, class A, B, and D carbapenemases have been identified in *P. aeruginosa*, and the class B MBL enzyme is the most prevalent (Queenan and Bush, 2007).

#### Class A Beta-Lactamases

The class A beta-lactamases include serine at amino acid (aa) 70 at the active site and the general base Glu-166 is involved in the catalytic process, which makes a difference from the other serine beta-lactamases of classes C and D (Matagne et al., 1999). In *P. aeruginosa*, the *Klebsiella pneumoniae* carbapenemase (KPC) and the Guiana extended-spectrum beta-lactamase (GES) belonging to the class A beta-lactamases with carbapenemase activity have been identified. The class A carbapenemases actively hydrolyze carbapenems and are partially inhibited by clavulanic acid.

**FIGURE 4:**
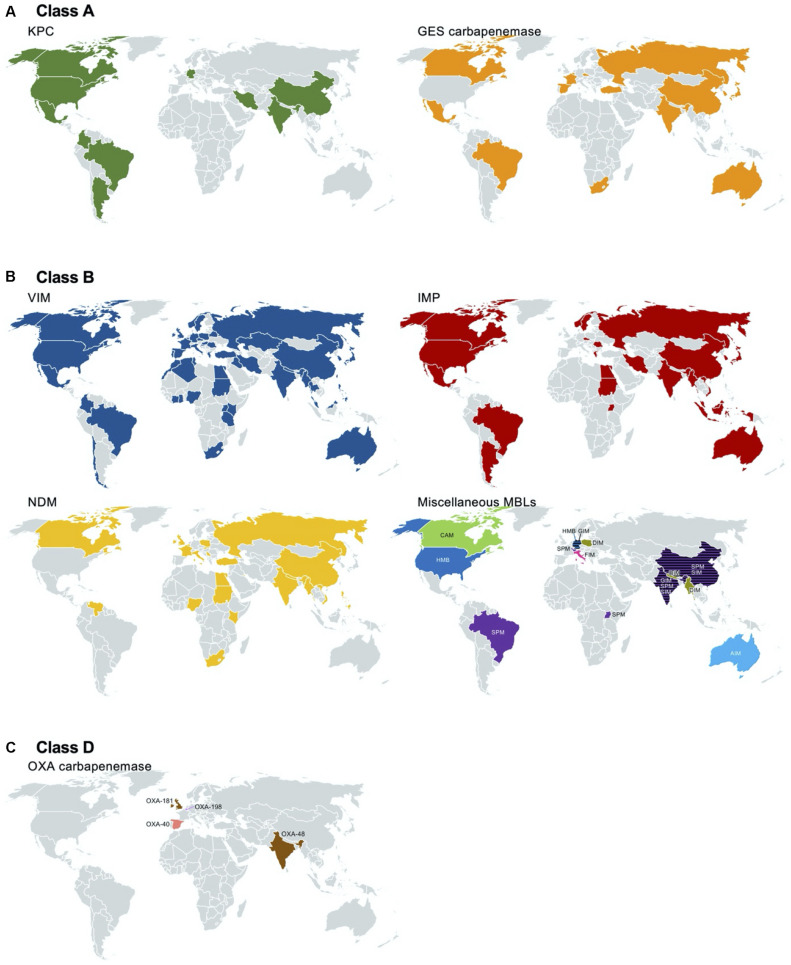
Worldwide identification of the carbapenemase-producing *Pseudomonas aeruginosa*. Reports of class A [*Klebsiella pneumoniae* carbapenemase (KPC) and Guiana extended-spectrum beta-lactamase (GES) with amino acid alteration at aa 170], class B [Verona integron-encoded MBL (VIM), imipenemase (IMP), New Delhi MBL (NDM), and others], and class D (OXA) carbapenemases are indicated in a geographic map. The references used for the map drawing are indicated in the main text.

*Klebsiella pneumoniae* carbapenemase was first discovered in a *K. pneumoniae* clinical isolate from North Carolina, United States, in 1996, presenting a specific pattern of resistance to penicillins, extended-spectrum cephalosporins, and aztreonam (Yigit et al., 2001). The first KPC-producing *P. aeruginosa* isolate was identified in Colombia in Villegas et al. (2007), and subsequent reports of the pathogen followed all over the world, including America (Akpaka et al., 2009; Poirel et al., 2010; Robledo et al., 2011; Jacome et al., 2012; Ramirez et al., 2013; Kazmierczak et al., 2016a; Walkty et al., 2019), Asia (Ge et al., 2011; Paul et al., 2015; Falahat et al., 2016; Hagemann et al., 2018), and Europe (Figure 4).

Among the GES-type class A beta-lactamases, only the variants with alterations at aa 170 from glycine to asparagine or serine with a polar uncharged side chain are able to hydrolyze carbapenems (Frase et al., 2009). Among the 43 GES variants, subtypes 4–6, 14–16, 18, 20, 21, 24, 25, 27–30, 33, 34, 36, 37, 39, 40–42, and 43 with serine at aa 170 and variants 2 and 13 with asparagine at aa 170 have carbapenem-hydrolyzing activity. Of note is that variant 22, having a substitution to leucine with a hydrophobic side chain at aa 170, does not confer resistance to carbapenems (Castanheira et al., 2014a). GES-type carbapenemase-producing *P. aeruginosa* has been identified in North and South America [Canada (McCracken et al., 2019), Mexico (Treepong et al., 2018), and Brazil (Polotto et al., 2012)], in Europe [including Belgium (Bebrone et al., 2013), Turkey (Malkocoglu et al., 2017), Spain (Viedma et al., 2009; Treepong et al., 2018), and Russia (Treepong et al., 2018)], in Africa [South Africa (Poirel et al., 2001); in the Middle East of Lebanon (Yaghi et al., 2019)], in Asia [including Japan (Hishinuma et al., 2018), India (Maurya et al., 2014), China (Wang et al., 2006a), and South Korea (Hong et al., 2016; Jabalameli et al., 2018)], and in Oceania [Australia (Sherry et al., 2018)] (Figure 4).

**FIGURE 5:**
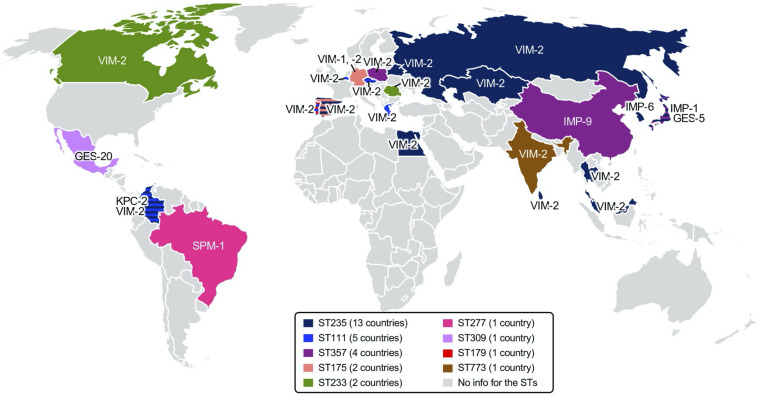
Epidemic carbapenemase-producing high-risk *Pseudomonas aeruginosa* clones identified worldwide. Regional dissemination of specific *P. aeruginosa* clones identified through multilocus sequence typing (MLST) is indicated in *color* on a geographic map, and the carbapenemases produced are indicated at the region. The number of countries with reports of the *P. aeruginosa* clone are indicated in the figure legends. References are indicated in the main text.

The carbapenem-hydrolyzing class A beta-lactamase producers mostly belong to ST235, ST111, ST357, and ST463. The KPC-, and GES-5-producing *P. aeruginosa* clones have been identified in Europe [Spain (Viedma et al., 2009), Italy (Giani et al., 2018), Lithuania (Mikucionyte et al., 2016), Turkey, Germany (Castanheira et al., 2014b), Belarus, and Russia (Edelstein et al., 2013)] and in Asian countries [Kazakhstan (Edelstein et al., 2013), Thailand (Khuntayaporn et al., 2019), China (Hu et al., 2015), and South Korea (Hong et al., 2016)] (Figure 5).

#### Class B Beta-Lactamases

The class B beta-lactamases are also known as “metallo-” beta-lactamases because they need divalent cations, usually Zn^2+^ ions, as a metal cofactor to hydrolyze beta-lactams. Although class B beta-lactamases are subclassified as B1, B2, and B3 based on structural and functional points (Frere et al., 2005), we will discuss subclass B1, the only dominant subclass in *P. aeruginosa*. The B1 subclass contains the largest number of clinically relevant acquired MBLs, not only in *P. aeruginosa* but also in Enterobacterales and other Gram-negative non-fermenters. MBLs bind two Zn^2+^ atoms for optimal hydrolysis. Zn^2+^ ion ligands bind at 3H (His–His–His) and DCH (Asp–Cys–His) sites, and the binding of di-Zn^2+^ plays a critical role in hydrolyzing beta-lactam substrates (Frere et al., 2005; Moran-Barrio et al., 2016). Consequently, the carbapenemase activity of MBLs is diminished in the presence of a chelator of Zn^2+^ and other divalent cations, i.e., ethylenediaminetetraacetic acid (EDTA). The substrate profile of the MBLs includes penicillins, cephalosporins, and carbapenems, but excludes monobactams. The acquired MBL genes, located mostly within a class 1 integron as gene cassettes, have been found in various bacterial species, including *P. aeruginosa*.

Metallo-beta-lactamase are the most prevalent type of carbapenemases produced by *P. aeruginosa* clinical isolates. VIMs are the most disseminated, followed by imipenemases (IMPs). New Delhi MBLs (NDMs) have also been identified. Regional dissemination of *P. aeruginosa* clinical strains producing Australian imipenemase (AIM), Central Alberta MBL (CAM), Dutch imipenemase (DIM), Florence imipenemase (FIM), German imipenemase (GIM), Hamburg MBL (HMB), São Paulo MBL (SPM), and Seoul imipenemase (SIM) was also reported.

A VIM-1-producing *P. aeruginosa* strain was first isolated in Verona, Italy, in Lauretti et al. (1999). Together with subtype VIM-2 (Poirel et al., 2000), the first two VIM enzymes were originally found in *P. aeruginosa* as gene cassettes of class 1 integrons. Notably, VIM-2 has a 10-fold more efficient hydrolyzing activity to both imipenem and meropenem compared to VIM-1 (Docquier et al., 2003), and in most of the countries, VIM-2 is the dominant carbapenemase produced by *P. aeruginosa* (Kazmierczak et al., 2016b). Thus far, a total of 66 variants of VIM have been identified, and subtype 2-producing *P. aeruginosa* has been extensively spread, with a few regional exceptions by specific outbreaks. VIM-producing *P. aeruginosa* clinical strains have been identified in almost all countries reporting surveillance data in six continents: in Europe (Poirel et al., 2000; Pournaras et al., 2002; Bahar et al., 2004; Deplano et al., 2007; Juan et al., 2008; Schneider et al., 2008; Siarkou et al., 2009; Bosnjak et al., 2010; Samuelsen et al., 2010; Mazzariol et al., 2011; Edelstein et al., 2013; Dortet et al., 2015; Kazmierczak et al., 2016b), in Asia (Wang et al., 2006b; Khosravi and Mihani, 2008; Nho et al., 2008; Al-Agamy et al., 2009; Castanheira et al., 2009; Azim et al., 2010; Khosravi et al., 2010; Koh et al., 2010; Mano et al., 2015; Kazmierczak et al., 2016b; Tada et al., 2017, 2019; Tohya et al., 2019; Yaghi et al., 2019), in North (Toleman et al., 2004; Pitout et al., 2007) and South America (Crespo et al., 2004; Pasteran et al., 2005; Kazmierczak et al., 2016a; Rios et al., 2018; Pacheco et al., 2019), in Africa (Pitout et al., 2008; Ktari et al., 2011; Jeannot et al., 2013; Moyo et al., 2015; Kateete et al., 2016; Adam and Elhag, 2018), and in Oceania (Tai et al., 2015; Figure 4). *P. aeruginosa* ST111 mostly produces VIM-2 and has been identified in European countries [the Czech Republic (Papagiannitsis et al., 2017), Portugal (Botelho et al., 2018), Italy (Giani et al., 2018), Greece, and Belgium (Castanheira et al., 2014b)] and in Latin American countries [Colombia (Correa et al., 2015)]. Less frequently, VIM-producing *P. aeruginosa* ST244 was identified in China (Chen et al., 2014; Feng et al., 2017), Brazil (de Oliveira Santos et al., 2019), and Africa (Cholley et al., 2014); VIM-producing *P. aeruginosa* ST175 was identified in Europe [Spain and Germany (Elias et al., 2010; Garcia-Castillo et al., 2011; Viedma et al., 2012; Giani et al., 2018)].

After the first IMP enzyme found in Japan in Watanabe et al. (1991), subsequent interspecies spread of the *bla*_IMP_ gene to Enterobacterales and *Acinetobacter baumannii* was reported in Japan and Europe, respectively (Arakawa et al., 1995). Currently, up to 79 variants of IMP have been identified, and varied subtypes have been identified with regional inconsistency. The global prevalent subtype is IMP-1, with a few exceptions of IMP-9 in China (Xiong et al., 2006) and IMP-6 in South Korea (Ryoo et al., 2009). Notably, IMP-6 has better hydrolyzing activity to meropenem than to imipenem compared to the other subtypes of IMP enzymes (Yano et al., 2001). IMP is the second most common carbapenemase produced by *P. aeruginosa* regardless of whether the strain was isolated in Europe (Hrabak et al., 2009, 2011; Samuelsen et al., 2010), in Asia (Xiong et al., 2006; Kouda et al., 2007; Nho et al., 2008; Ryoo et al., 2009; Azim et al., 2010; Khosravi et al., 2010; Koh et al., 2010; Tada et al., 2019; Yaghi et al., 2019), in North (Gibb et al., 2002; Hanson et al., 2006), and South America (Garza-Ramos et al., 2008; Kazmierczak et al., 2016b), in Africa (Kateete et al., 2016; Adam and Elhag, 2018), and in Oceania (McCarthy et al., 2017; Figure 4). *P. aeruginosa* ST357 mostly produces IMP. The clone was spread in Europe (Hrabak et al., 2011; Papagiannitsis et al., 2017) and Asia (Kouda et al., 2009; Fan et al., 2016). IMP-producing *P. aeruginosa* ST621 was identified in Europe [Italy (Giani et al., 2018) and France (Fournier et al., 2012)] (Figure 5).

The NDM enzyme was first identified in 2008 in *K. pneumoniae* isolated from a Swedish patient with a history of travel to India (Yong et al., 2009). Shortly after, the NDM producers became endemic in South Asian countries located in the Indian subcontinent, such as India, Pakistan, and Bangladesh (Kumarasamy et al., 2010; Dortet et al., 2014). The first report of NDM-1-producing *P. aeruginosa* was in Serbia in Jovcic et al. (2011), and subsequent dissemination was observed in the Balkans (Jovcic et al., 2013; Kulkova et al., 2015). NDM-producing *P. aeruginosa* has been identified mostly in Asia (Khajuria et al., 2013; Paul et al., 2015; Tada et al., 2017, 2019; Honda et al., 2019; Tohya et al., 2019), in Europe (Jovcic et al., 2011; Carattoli et al., 2013; Janvier et al., 2013; Kulkova et al., 2015; Urbanowicz et al., 2019), and in Africa (Manenzhe et al., 2015; Kateete et al., 2016; Adam and Elhag, 2018). And, as a sporadic emergence, it was also identified in North America (Mataseje et al., 2016; Figure 4). Among a total of 27 variants of NDM that have been identified, NDM-1 seems to be the most prevalent subtype.

Being different from the intercontinental dissemination of VIM-, IMP-, and NDM-producing *P. aeruginosa*, other MBL-producing *P. aeruginosa* outbreaks or regional spreading have occurred, except for SPM-producing *P. aeruginosa*. SPM-producing *P. aeruginosa* was first identified in São Paulo, Brazil (Toleman et al., 2002), and the clone belonged to ST277 (Chaves et al., 2017; de Oliveira Santos et al., 2019). The SPM producer was subsequently identified in Switzerland (Salabi et al., 2010), the United Kingdom (Salabi et al., 2010), India (Azim et al., 2010), China (Cai et al., 2016), and Uganda (Kateete et al., 2016; Figure 4). DIM-producing *P. aeruginosa* clinical strains were regionally disseminated mostly in Asia (Tada et al., 2017, 2019) and Europe (Urbanowicz et al., 2019). GIM-producing *P. aeruginosa* was identified in Germany (Castanheira et al., 2004; Wendel et al., 2015) and India (Azim et al., 2010). SIM-producing *P. aeruginosa* was identified in China (Sun et al., 2016). HMB-producing *P. aeruginosa* was identified both in Hamburg, Germany (Pfennigwerth et al., 2017), and in the United States (Walters et al., 2019). CAM-producing *P. aeruginosa* was identified in Canada (Boyd et al., 2019), and no more dissemination was observed. The AIM and FIM producers were restricted in Australia (Yong et al., 2012) and Italy (Pollini et al., 2013), respectively (Figure 4).

#### Class D Beta-Lactamases

Class D beta-lactamases belong to the superfamily of serine beta-lactamases with a unique carboxylated Lys-73 responsible for the beta-lactam hydrolysis activity (Golemi et al., 2001). The carbapenem-hydrolyzing class D beta-lactamases (CHDLs) were first described in *A. baumannii* and published by Paton et al. (1993). CHDLs are serine beta-lactamases with a relatively weak activity against carbapenems and are poorly inhibited by EDTA or clavulanic acid. Among a total of 12 groups of CHDLs, three groups—OXA-40-like, OXA-48-like, and OXA-198-like—have been identified in *P. aeruginosa*.

OXA-type carbapenemases are rarely identified in *P. aeruginosa*, and the emergence of the following strains has been reported: OXA-40-producing *P. aeruginosa* in Spain (Sevillano et al., 2009), OXA-48-producing *P. aeruginosa* in India (Borah et al., 2016), OXA-181-producing *P. aeruginosa* in the United Kingdom (Findlay et al., 2017), and OXA-198-producing *P. aeruginosa* in Belgium (Bonnin et al., 2018; Figure 4).

## Mobile Genetic Elements Associated With Carbapenemase-Encoding Genes

The acquired genes encoding carbapenemases are associated with a plethora of mobile genetic elements, such as plasmids, gene cassettes of integrons, transposons, and genomic islands (Kung et al., 2010). Mobile genetic elements have the ability to move from genome to genome by transformation, conjugation, and transduction, presenting intracellular and intercellular mobility (Roberts et al., 2008).

### *Pseudomonas aeruginosa* Plasmids Carrying the Carbapenemase-Encoding Genes

In general, the plasmids carrying the carbapenemase-encoding genes in *P. aeruginosa* belong to the distinct incompatibility groups from those in Enterobacterales. Among the 13 known incompatibility types of IncP, IncP-2-type plasmids are classic types frequently identified in *P. aeruginosa* (Korfhagen et al., 1976). Among the 207 complete genomes of *P. aeruginosa* of the Genome Database (NCBI, 2020), five genomes include a plasmid carrying one or two carbapenemase-encoding genes; two of the five plasmids are of the IncP-2 incompatibility type, while the other three are untypable: two *bla*_KPC–2_ genes are harbored by untypable plasmids, which are almost the same (identical nucleotide sequences, except for a 1-bp gap difference between the 57,053- and 57,052-bp plasmids), one *bla*_VIM–1_ is harbored by an untypable plasmid, one IncP-2 plasmid harbors *bla*_IMP–45_, and one IncP-2 plasmid harbors both the *bla*_VIM–1_ and the *bla*_IMP–45_ genes.

Among the plasmids in *P. aeruginosa* having an incomplete genome, the 31,529- and 38,939-bp IncP-6 plasmids carrying the *bla*_KPC–2_ gene have been identified in Colombia and China, respectively (Naas et al., 2013; Dai et al., 2016), and the 7,995-bp IncU plasmid including the *bla*_KPC–2_ gene has been identified in Colombia (Naas et al., 2013). An untypable 3,652-bp plasmid harboring the *bla*_KPC–2_ gene was identified in Brazil (Galetti et al., 2016).

Recently, a *Pseudomonas* plasmid lineage carrying the MBL genes has been reported (Di Pilato et al., 2019). A retrospective analysis revealed that the plasmid lineage has been identified since the 1990s, mostly in Europe. While the plasmid does not belong to a recognized plasmid type, the type 4 secretion system components classified the plasmids as MOBF11 or MPFT plasmid families. The MBL genes in the plasmid were identified as gene cassettes of the class 1 integron In*70* (Di Pilato et al., 2019).

### Carbapenemase-Encoding Gene-Associated Transposable Units

**FIGURE 6:**
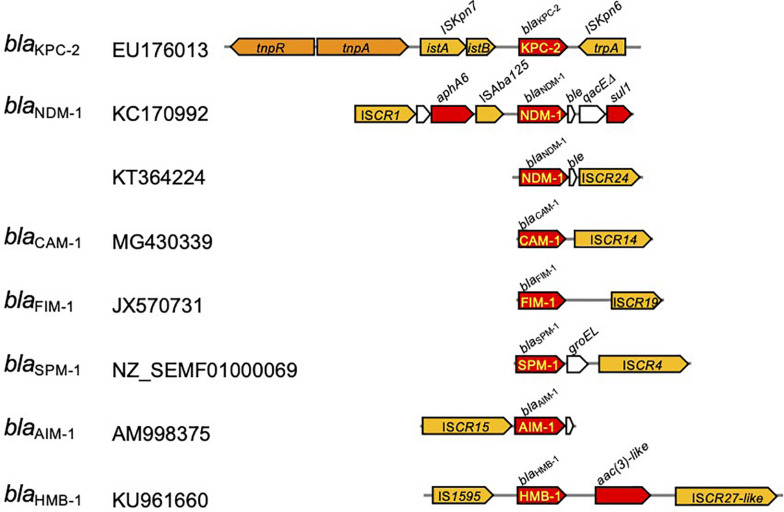
Transposable units identified in *Pseudomonas aeruginosa* carrying the carbapenemase-encoding genes. The *red arrow* indicates the genes for antimicrobial resistance, and those with *yellow letters inside* indicate the genes for carbapenemase. *Yellow arrows* depict insertion sequences, and those in *orange* indicate transposase/resolvase.

**FIGURE 7:**
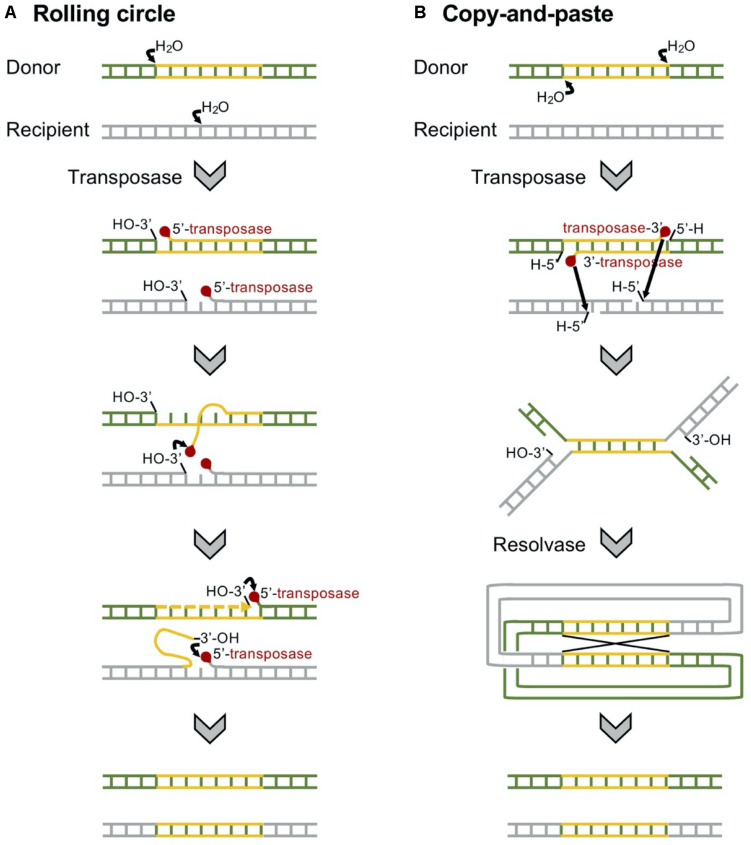
Mechanisms of replicative transposition of the transposons. **(A)** Insertion sequence with a common region (IS*CR*)-mediated rolling circle replicative transposition is involved in rolling circle replication. IS*CR* elements lack terminal inverted repeats, and a single copy of the element is able to transpose adjacent DNA sequences (Toleman et al., 2006). **(B)** A Tn*3*-mediated copy-and-paste replicative transposition requires both a transposase and a resolvase. The transposon is replicated, joining the donor and the recipient in a cointegrate, which is resolved to give the donor and the recipient of the transposon (Grindley, 1983).

The carbapenemase-encoding genes are frequently included in transposable elements, which are often associated with the insertion sequences with a common region (IS*CR*s) being responsible for the rapid transmission of bacterial multidrug resistance (Figure 6; Toleman et al., 2006). Typically, the IS*CR* element lacks flanking inverted repeats (IRs) and the integration does not produce direct repeat (DR) sequences (Diaz-Aroca et al., 1987). Rolling circle has been suggested for the transmission mechanism of IS*CR*s (Figure 7), and the transposition method allows a polarized transfer of the IS*CR* elements to mobilize adjacent DNA sequences in varied sizes (del Pilar Garcillan-Barcia et al., 2001).

The *bla*_CAM_ and *bla*_FIM_ genes were identified in transposable elements associated with IS*CR14* and IS*CR19*, respectively, located downstream from the gene. The *bla*_SPM_ gene was identified in *P. aeruginosa* accompanied with the chaperone *groEL* gene and flanked by a pair of IS*CR4* elements. The *bla*_AIM_ gene was identified in a transposable element associated with the upstream IS*CR15*. The *bla*_HMB__–1_ gene was in a transposable unit, namely, Tn*6345* (Supplementary Table 1; Pfennigwerth et al., 2017), flanked by IS*1595* upstream and IS*CR27*-like downstream (Toleman et al., 2006). For the *bla*_NDM_ gene, the composite transposon Tn*125* flanked by a pair of IS*Aba125* copies at both ends has been identified in *A. baumannii* (Bontron et al., 2016). The *bla*_NDM__–1_ gene-associated transposable element is composed of a truncated Tn*125*. IS*CR* is located upstream from the ΔIS*Aba1*, and downstream of the *bla*_NDM–1_ gene, the *ble* gene for bleomycin resistance and the *trpF* gene for phosphoribosylanthranilate isomerase are followed (Sole et al., 2011).

The Tn*4401* carrying the *bla*_KPC_ gene has been found in diverse bacterial hosts, including *P. aeruginosa* (Cuzon et al., 2011; Figure 6). Tn*4401* is a 10-kb transposon composed of genes encoding a transposase and a resolvase, and the *bla*_KPC_ gene together with two insertion sequences (ISs), IS*Kpn6* and IS*Kpn7* (Figure 6), and transposition of Tn*4401* occurs through the mechanism of copy-and-paste replicative transposition (Figure 7; Grindley, 1983). Tn*4401* includes nine isoforms from a to I differing in the sequences upstream of the *bla*_KPC_ gene (Naas et al., 2012; Bryant et al., 2013; Cheruvanky et al., 2017; Araujo et al., 2018; Schweizer et al., 2019). While both Tn*4401a* and Tn*4401b* are prevalent in Enterobacterales (Stoesser et al., 2017; Yoon et al., 2018), Tn*4401b* is the only isotype identified in *P. aeruginosa*. Seven Tn*4401* elements were found through the restricted BLAST against the species *P. aeruginosa*, and all were Tn*4401b*: two Tn*4401b* copies in a chromosome (GenBank accession CP029605) and each Tn*4401b* copy in five plasmids (GenBank accessions MN082782.1, CP027168.1, CP029092.1, KC609323.1, and EU176013.1).

### Class 1 Integrons Carrying Carbapenemase-Encoding Gene Cassettes

**FIGURE 8:**
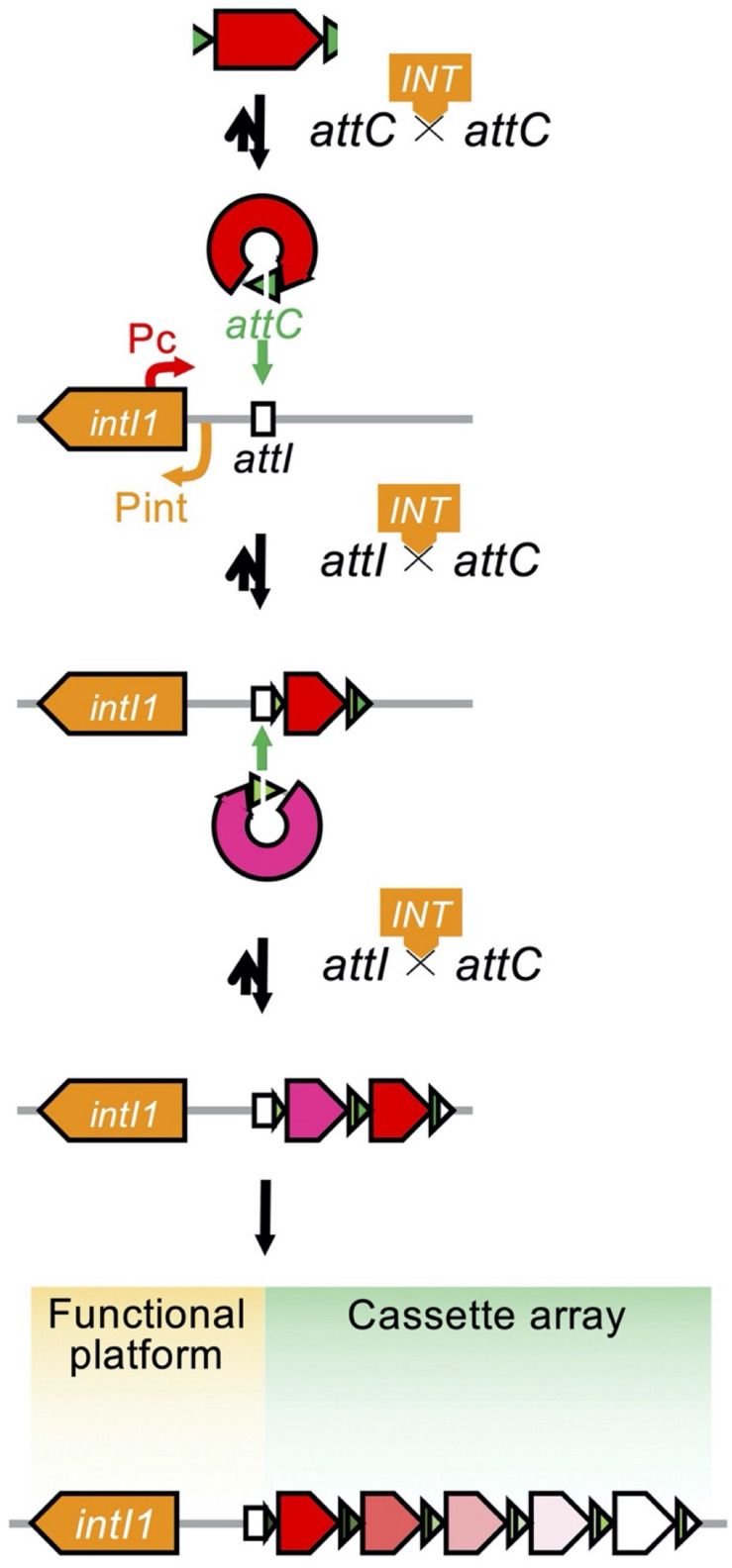
Organization of the integron and the process of capturing and exchanging the gene cassettes. The *open arrow* indicates the open reading frame and is colored based on its function: the antimicrobial resistance gene is in *red* or in *magenta*, the *attC* gene is in *green*, and the *intI1* gene is in *orange*. The *open box* indicates the *attI* locus. The integron is organized as a functional platform including the *intI1* gene and the *attI* locus and a cassette array assembled through the acquisition of gene cassettes structured with an open reading frame and an *attC* locus. Expression of the gene cassettes is dependent on the common promoter, Pc, and the level of expression depends on the distance from the Pc. The IntI1 integrase binds to the *attC* locus of the excised gene cassette to help circularize the cassette (Collis et al., 1993; Hall and Collis, 1995).

Integrons are assembly platforms comprising an *intI* gene for a site-specific tyrosine recombinase, an *attI* for a primary recombination site, the promoter Pc for transcription, and an assemblage of passenger genes composing a gene cassette array (Figure 8; Collis et al., 1993). The IntI integrase recognizes the *attC* site of the gene cassette and the promoterless gene cassette is inserted as a linear form in the integron (Figure 8; Collis and Hall, 1992). In addition to the catalysis of *attC* × *attC*, the integrase catalyzes *attI* × *attC*, leading to the gene cassette integration into the *attI* site. A successive integration of the gene cassettes occurs downstream of the resident Pc promoter (Hall and Collis, 1995). The expression of the gene cassette is dependent on the sole promoter and the level of gene expression depends on the distance from the sole promoter Pc (Coyne et al., 2010). By nature, the first few cassettes are expressed and the rest of the array exists as a reservoir of standing genetic variation (Cambray et al., 2010). Rearrangement of the order of gene cassettes affects the resistance phenotype of the bacterial host (Hall and Collis, 1995; Rowe-Magnus et al., 2002).

#### Carbapenemase-Encoding Gene Cassettes

The class A carbapenemase-encoding gene *bla*_GES_, the MBL genes *bla*_VIM_, *bla*_IMP_, and *bla*_GIM–1_, and the class D gene *bla*_OXA–198_ were identified as a gene cassette composing a class 1 integron. In the integron database INTEGRALL (^4^ last updated on 10 December 2020) (Moura et al., 2009), a total of 812 class 1 integrons of 282 different gene cassette arrays were identified for the organism *P. aeruginosa*. Among them, 191 class 1 integrons of 148 different arrays carried one or two carbapenemase-encoding gene cassettes (Supplementary Table 2). The most prevalent *bla*_VIM_ genes were identified as gene cassettes in 98 arrays of the 130 class 1 integrons. Among them, 13 carried only the *bla*_VIM_ cassette; the others harbored additional gene cassettes encoding aminoglycoside-modifying enzymes, mostly the *aacA* genes and less frequently the *aadA* or *aadB* genes. All but five carried the *bla*_VIM_ gene cassette in the first position of the array (62.3%) or in the second position of the array (33.1%).

The second most dominant *bla*_IMP_ cassettes were identified in 50 class 1 integrons of 40 different arrays, and 14 of the integrons harbored a single gene cassette. All but two harbored the *bla*_IMP_ gene cassette in the first of the array (70.0%) or in the second of the array (22.9%). Less frequently, the *bla*_GIM–1_ gene cassette was identified in six class 1 integrons of five different arrays all in the first of the array, and three *bla*_GES–5_ cassette-associated and one of each of the *bla*_GES–8_, *bla*_GES–9_, and *bla*_GES–15_ cassette-associated class 1 integrons were identified.

#### Mobilized Class 1 Integrons

**FIGURE 9:**
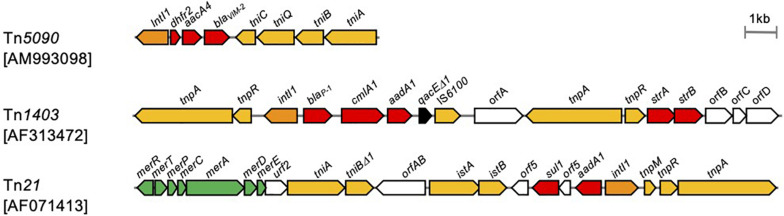
Transposons giving mobility to integrons. Transposons are indicated in scale with the component. The accession number of each sequence is indicated in *brackets*. *Arrows* indicate open reading frames with *filled colors* that differ by function: *yellow*, transposition; *orange*, class 1 integrase; *red*, antimicrobial resistance; *green*, heavy metal resistance (Radstrom et al., 1994; Minakhina et al., 1999; Shi et al., 2018).

Basically, class 1 integrons are immobile per se, and a set of functional transposition modules is needed for transposition (Martinez et al., 2012). The Tn*402* family transposon Tn*5090* is a good example of a functional transposon giving mobility to a class 1 integron (Figure 9; Radstrom et al., 1994). The Tn*402* family transposon is a Mu-related transposon that has been identified in the broad-host IncP plasmid R751 conferring trimethoprim resistance (Jobanputra and Datta, 1974; Shapiro and Sporn, 1977). The transposon family has two modules: a transposition module composed of the transposase TniA, the ATP-binding protein TniB, the transposition auxiliary protein TniQ-resolution site *res*, the serine resolvase TniC, and a class 1 integron carrying antimicrobial resistance gene cassettes. Transposition of the Tn*402* family transposon involves a TniABQ-dependent cointegrate formation and a site-specific serine resolvase-dependent resolution (Radstrom et al., 1994). The transposon has 25-bp inverted repeats, and the integration generates 5-bp DRs. The transposon targets the *res* site of Tn*21* subfamily transposons as well as resolution sites found on plasmids (Minakhina et al., 1999). The Tn*21*-like Tn*1403*, Tn*6060*, Tn*6162*, and Tn*6249*, which nest inside the Tn*402*-like, carry a class 1 integron possessing the *bla*_VIM–1_ gene cassette (Di Pilato et al., 2015).

In the transposon repository database (Tansirichaiya et al., 2019), a total of 20 transposons carried the gene encoding carbapenemases (Supplementary Table 1), and of those, 19 transposons are associated with class 1 integrons harboring the *bla*_VIM_ (*n* = 9), *bla*_IMP_ (*n* = 6), *bla*_GES_ (*n* = 2), *bla*_SIM_ (*n* = 1), and *bla*_DIM_ (*n* = 1) gene cassettes. The 207 complete *P. aeruginosa* genomes from the Genome Database included a total of 29 chromosomal class 1 integrons with a carbapenemase-encoding gene cassette. Among those, 20 integrons included a part or the entire set of the TniABQR transposition module of the Tn*402*-like.

### Resistance Islands Harboring the Carbapenemase-Encoding Genes

Genomic islands carrying many foreign genes are useful for adaptation by providing multiple fitness-associated elements to *P. aeruginosa* in a single event of horizontal gene transfer (Kung et al., 2010). Genomic islands are often free to move in and out of the chromosome, and pKLC102, a 100-kb plasmid identified in *P. aeruginosa* C strain, is an example of this (Klockgether et al., 2004). The plasmid pKLC102 was found simultaneously in the chromosome as a genomic island being integrated into the 3′ end of the tRNA^*Lys*^ gene in favor of an *att* site.

Genomic islands are typically inserted at the 3′ end of a transfer RNA (tRNA) gene; however, they are also targeted elsewhere in the chromosome (Klockgether et al., 2011). Genomic islands are easily differentiated from the core genome by their atypical G+C contents, differing from the typical *P. aeruginosa* G+C content of 65–67% and atypical oligonucleotide usage. Genomic islands typically harbor the genes for factors involved in mobility, such as integrases, transposases, ISs, and other components responsible for biological processes. Genomic islands are categorized accordingly to their main characteristics determined by the gene content, such as pathogenicity, symbiosis, metabolic, fitness, or drug resistance. Thus far, 25 genomic islands have been identified: two *P. aeruginosa* pathogenic islands, PAPI-1 and PAPI-2; 17 *P. aeruginosa* genomic islands, PAGI-1 to PAGI-17; five Liverpool epidemic strain genomic islands, LESGI-1 to LESGI-5; and a plasmid-origin genomic island, pKLC102. As an effort to group the genomic islands, Kung et al. (2010) suggested two families of genomic islands by conserved function and synteny of the backbone genes, and Klockgether et al. (2011) proposed that any known genomic islands originated from an ancestry based on conserved orthologs. However, no scheme is publicly endorsed.

Genomic islands carrying the antimicrobial resistance genes, so-called resistance islands, specifically provide a great advantage for survival to bacterial hosts in clinical settings. Among the 25 genomic islands, only three—PAGI-13 in *P. aeruginosa* ST277 (Silveira et al., 2014) and PAGI-15 in ST244 and PAGI-16 in ST235, respectively (Hong et al., 2016)—were resistance islands carrying antimicrobial resistance determinants, and only two, PAGI-15 (*bla*_GES–24_) and PAGI-16 (*bla*_IMP–6_, or *bla*_IMP–10_), harbored a carbapenemase-encoding gene (Table 1; Hong et al., 2016). One unnamed PAGI-2-like island in *P. aeruginosa* ST235 carrying the *bla*_GES–5_ gene has also been reported (Abril et al., 2019).

**TABLE 1:** Designated *Pseudomonas aeruginosa* genomic islands harboring the antimicrobial resistance genes.

Genomic island	Length (bp)	GC (%)	CDS(*n*)	Antimicrobial resistance determinants	Heavy metal resistance determinants	Virulence determinants	Function	Phage	GenBank accession
PAGI-13	197,350	62.3		*bla*_OXA–56_, *sul1* (x2), *aac(6′)-Ib*, *aadA7*, *rmtD*	*–*	–	Resistance to multiple antimicrobials	–	KT454971
PAGI-15^a^	118,715	61.3	109	*bla*_GES–24_, *bla*_OXA–2_, *aac(6′)-Ib-cr, ant(2″)-Ia, aph(3″)-Ib, aph(6)-Id, tet*(G), *sul1*	*merA*	T4SS	Resistance to multiple antimicrobials and mercury. Expression of a type IV secretion system	*Pseudomonas* phage Pf1	KX196168
PAGI-16	95,909	61.4	86	*bla*_IMP–10_, *bla*_OXA–1_, *aac(6′)-Ib-cr, aadA1, sul1, catB3*	*merA*	T4SS	Resistance to multiple antimicrobials and mercury. Expression of a type IV secretion system	–	KX196169

*^a^PAGI-15 is assigned as transposon Tn6534 by The Transposon Registry.*

In addition, the PA143/97 genomic island, which was manufactured to include the Tn*6249* carrying two class 1 integrons harboring the *bla*_VIM–2_ gene cassette, was reported (Martinez et al., 2012; Di Pilato et al., 2015).

*Pseudomonas aeruginosa* is able to obtain multidrug resistance at once through resistance islands. Since this review focuses on carbapenem resistance in *P. aeruginosa*, the exploration is restricted to genomic islands harboring carbapenemase-encoding genes.

Among the 207 complete genomes of *P. aeruginosa* extracted from the Genome Database, a total of 38 chromosomes harbor resistance islands carrying the carbapenemase-encoding gene, and eight of those carry two resistance islands. In total, 45 resistance islands, sized between 8,858 and 117,103 bp, were analyzed. The G+C contents varied from 55.5 to 65.5%, which were lower than those of the chromosomes, which were from 65 to 67%, and the integration sites varied.

**FIGURE 10:**
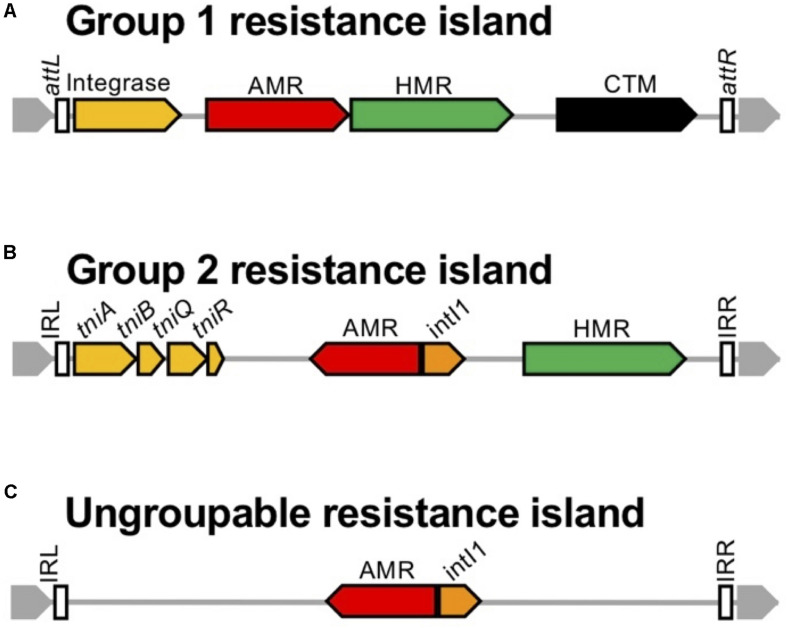
Schematic presentation of the structure of genomic islands belonging to the three groups. The schematic structure is presented for the three groups of genomic islands: **(A)** Group 1 resistance islands equipping the tyrosine-based integrase gene at the 5’ terminal and the conjugative transfer machinery gene cluster at the 3’ terminus, **(B)** Group 2 resistance islands carrying a transposition module of a whole or a partial TniABQR component, **(C)** and the others. The genes are indicated with *open arrows* and colored based on function: genes for integration and recombination of the genomic island are in *yellow*, genes for antimicrobial resistance (AMR) are in *red*, genes for heavy metal resistance (HMR) are in *green*, the *intI1* gene is in *orange*, conjugative transfer module mostly composed of the type 4 pili assembly genes is in *black*, and the core gene is in *gray*. The *open box* indicates inverted repeats (IRs) left and right, named the *attL* and *attR* loci for integration conjugative elements.

The 45 resistance islands carrying the carbapenemase-encoding gene were classified into three groups by the transposition module (Table 2). Group 1 resistance islands furnish the tyrosine-based integrase gene at the 5’ terminal and the conjugative transfer machinery gene cluster at the 3′ terminus (Figure 10). Group 1 can be further grouped into two subgroups: group 1a, with a core structure of ICE_Tn_*_4371_* mostly targeting OprD of OccD4/OpdT tyrosine or adenylate cyclase ExoY, and group 1b, which resembles the known multidrug-resistant genomic islands PAGI-13, PAGI-15, and PAGI-16 targeting tRNA_Gly_. Resistance islands belonging to group 1a had G+C contents between 64.5 and 65.1%, and the size was 44–74 kbp. The *bla*_SPM–1_ and *bla*_NDM–1_ genes carried by the group 1a resistance islands were all associated with IS*91*-like IS*CR*s composing unit transposons, and neither the other antimicrobial resistance determinants nor the heavy metal resistance-associated genes were identified in the resistance island. Meanwhile, the G+C contents of group 1b ranged between 60.5 and 64.2%, and the size ranged from 37 to 117 kbp. The upper cluster of group 1b, including four genomic islands (Table 2), targeted tRNA_Gly_ at the locus PA0714 of the genome of *P. aeruginosa* PAO1 (NCBI RefSeq, NC_002516.2). Characteristically, the genomic islands belonging to the cluster always possess the mercury resistance gene cluster, which is likely derived from Tn*501*, and the carbapenemase-encoding genes are carried by class 1 integrons as a gene cassette. The eight genomic islands belonging to the next cluster of subgroup 1b targeted tRNA_Gly_ either at the locus PA2583 or PA2817 of the PAO1 genome. A quarter of the genomic islands possess a class 1 integron, and the acquisition of the carbapenemase-encoding gene is mostly due to IS*CR* elements. Pieces of the heavy metal gene cluster have been observed.

**TABLE 2:**
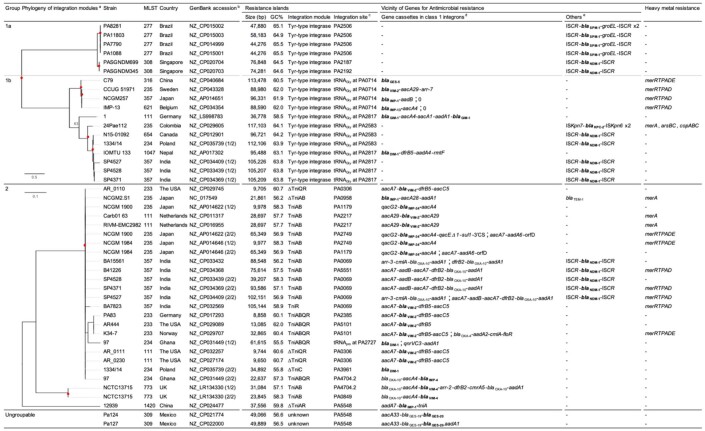
Resistance islands carrying the carbapenemase-encoding gene in *Pseudomonas aeruginosa*.

*^a^The molecular phylogenetic trees were constructed using the aligned amino acid sequences of the integration module, either the tyrosine-type integrase/recombinase or the concatenated TniABQR for transposition, using the maximum likelihood method implemented in the PhyML program (v. 3.0 aLRT) with the WAG matrix and a gamma correction for variable evolutionary rates (Guindon and Gascuel, 2003). A total of 100 bootstrap experiments are carried out; the red dots at the node indicate perfect robustness.^b^If the chromosome includes two genomic islands, each case is indicated with numbers 1/2 and 2/2 in brackets following the GenBank accession number.^c^Integration sites are indicated using the locus tag of the genome of P. aeruginosa PAO1 (NCBI RefSeq, NC_002516.2).^d^In0 is indicated with “0.”^e^Antimicrobial resistance genes located in a class 1 integron and transposons are indicated, and the genes encoding carbapenemase are in boldface.*

The group 2 resistance islands contain a transposition module composed of a whole or a partial TniABQR component. The resistance islands had obviously lower G+C contents, between 55.5 and 62.0%, and the sizes were diverse, from 8.8 to 105 kbp. The composition of the resistance islands in group 2 corresponds to that of the *Proteus* genomic island PGI-1 (Mac Aogain et al., 2016). The resistance islands are constructed through the accumulated assemblage on a Tn*402* backbone. Such an assemblage of transposons is commonly observed in the AbaR-type resistance islands in *A. baumannii*, which evolved from the Tn*6019* backbone (Krizova et al., 2011).

In the case of the remaining two resistance islands, categorization is unavailable since only two cases of possible clonal relations are available from the GenBank database. Neither a specific tyrosine-based recombinase nor a transposition component has been identified in the resistance islands; however, they are flanked by 20-bp inverted repeats.

## Conclusion

Carbapenems represent a valuable therapeutic option for patients with infections caused by multidrug-resistant *P. aeruginosa*. It is ironic that carbapenem resistance, especially that conferred by carbapenemase production, is closely related to multidrug resistance, highlighting the role of modular mobile units carrying multiple antimicrobial resistance determinants. The molecular epidemiology of antimicrobial resistance has been studied by traditional methodologies based on PCR and Sanger sequencing to identify the resistance genes and to distinguish the fundamental mobile genetic elements carrying the gene, i.e., gene cassettes of integrons, transposons, and plasmids. The present era of massive next-generation sequencing, mostly the long-read sequencing, allows resolving a wide range of complex genome regions, such as modular mobile units associated with genes for antimicrobial resistance, also known as resistance islands. Such an extensive analysis has been carried out for limited numbers of the *P. aeruginosa* genome mostly for the genomic islands unrelated to antimicrobial resistance determinants. Among the plenty genomic islands in the genome of *P. aeruginosa*, resistance islands have a meaning beyond genome plasticity. Such a modular mobile unit harboring antimicrobial resistance determinants is able to disseminate by itself and capture an alien gene for resistance, which means the resistance islands and high-risk clones are the A to Z of acquisition of multidrug resistance. Our trial to classify *P. aeruginosa* resistance islands needs improvement with more cases for resistance islands.

The global spread of the carbapenem-resistant *P. aeruginosa* is one of the major global public health challenges, and the epidemiological scenario is often associated with the circulation of carbapenemase-encoding genes linked with (i) the endemic carbapenemase gene and (ii) the carbapenem usage in clinical settings. The KPC-producing *P. aeruginosa* in KPC-endemic United States, the SPM-producing *P. aeruginosa* in SPM-endemic Brazil, and the NDM-producing *P. aeruginosa* in NDM-endemic India exemplify well the first linkage. The second linkage is illustrated through the dominance of IMP-6-producing *P. aeruginosa* in South Korea, in which meropenem usage is approximately twice more in clinical settings than that of imipenem. Since IMP-6 has greater hydrolyzing activity to meropenem than to imipenem compared to the other subtypes of IMP enzymes, producing the IMP-6 subtype is favorable to the bacterial host. It emphasizes that the carbapenemase-producing organisms should be controlled regardless of the bacterial host, and control includes both surveillance study and antimicrobial stewardship. Needless to say, more attention is needed to be paid to the emergence and spread of the high-risk *P. aeruginosa* clones, together with their enzymatic/non-enzymatic carbapenem resistance. Though continuing efforts are being made to develop the beta-lactamase inhibitors in order to preserve the efficacy of beta-lactam drugs including carbapenems, it is fruitful just for the serine beta-lactamases. Development of the inhibitors for MBLs, which are frequently produced by *P. aeruginosa*, is eager to be accelerated.

Despite the efforts to control the spread of carbapenem-resistant *P. aeruginosa*, a conclusive solution to the issue is still far from being accomplished. Surveillance study for the drug-resistant pathogen is essential and global collaboration using harmonized methods is important for a practical comparison of the outputs. In addition, to fight against the drug-resistant pathogen, we need to understand how the pathogens acquire resistance determinants. Taking the advantages of up-to-date techniques, assessment of the bacterial genome should be carried out, not only for the mobile genetic elements carrying a carbapenemase-encoding gene but also for the genomic islands. Furthermore, through the technique, the mobile genetic elements should be investigated extensively, and it would allow a comprehensive grasp of the dissemination of drug resistance.

## Author Contributions

SJ conceived and supervised the study, performed the data evaluation and confirmation, and finalized and edited the manuscript. E-JY carried out the analysis and data validation, and drafted the manuscript. Both authors contributed to the article and approved the submitted version.

## Conflict of Interest

The authors declare that the research was conducted in the absence of any commercial or financial relationships that could be construed as a potential conflict of interest.

## Footnotes

1 https://www.ncbi.nlm.nih.gov/pathogens/antimicrobial-resistance/

2 https://www.ncbi.nlm.nih.gov/pathogens/isolates#/refgene/

3 https://www.ncbi.nlm.nih.gov/genome/

4 http://integrall.bio.ua.pt/
