# Supplementary material for: Mobile Carbapenemase Genes in Pseudomonas aeruginosa
Source: Front Microbiol. 2021 Feb 18;12:614058. doi: 10.3389/fmicb.2021.614058 (PMC7930500; doi:10.3389/fmicb.2021.614058)
Supplement: Supplementary file 1 [file Table_1.DOCX]

**Table S1** Carbapenemase transposons identified in the *P. aeruginosa* chromosome or plasmids deposited in The Transposon Registry [182]

| Transposon | Type | Size (kb) | Accession | Carbapenemase gene | Extra function |
| --- | --- | --- | --- | --- | --- |
| Tn*6001*^192^ | Unit transposon | 12.1 | EF138817.1 | *bla*_VIM-3_ | - |
| Tn*6016*^193^ | Unit transposon | 8.8 | KC543497.1 | *bla*_IMP-9_ | Heavy metal resistance |
| Tn*6060*^194^ | Composite transposon | 25.4 | GQ161847.1 | *bla*_VIM-1_ | - |
| Tn*6163*^195^ | Unit transposon | 21.2 | JF826499.1 | *bla*_GES-5_ | Heavy metal resistance |
| Tn*6217*^193^ | Unit transposon | 6.9 | KC543497.1 | *bla*_IMP-9_ | - |
| Tn*6249*^196^ | Composite / Unit transposon | 26 | LK054503.1 | *bla*_VIM-1_ | - |
| Tn*6284*^197^ | Unit transposon | 22.6 | KU254577.1 | *bla*_SIM-2_ | Heavy metal resistance |
| Tn*6286**^198^ | Unit transposon | 23.7 | KU130294.1 | *bla*_DIM-2_ | - |
| Tn*6292*^199^ | Unit transposon | - | - | *bla*_IMP-4_ | - |
| Tn*6345* | Unit transposon | 6.0 | KU961660.2 | *bla*_HMB-1_ | - |
| Tn*6352*^200^ | Unit transposon | 11.0 | KX889311.1 | *bla*_VIM-2_ | - |
| Tn*6356*^201^ | Unit transposon | 15.6 | KY494864.1 | *bla*_VIM-2_ | Efflux pump |
| Tn*6391* | Unit transposon | 17.4 | MF168946.1 | *bla*_VIM-2_ | Heavy metal resistance |
| Tn*6392*^202^ | Unit transposon | 14.5 | MF144194.1 | *bla*_VIM-24_ | Heavy metal resistance |
| Tn*6394*^203^ | Unit transposon | 7.2 | MF344578.1 | *bla*_IMP-1_ | - |
| Tn*640*^202^ | Composite / Unit transposon | 30.0 | CP030075.1 | *bla*_VIM-4_ | Heavy metal resistance |
| Tn*6411*^203^ | Composite / Unit transposon | 37.6 | CP024477.1 | *bla*_IMP-1_ | - |
| Tn*6413*^202^ | Conjugative transposon | 114.1 | CP030075.1 | *bla*_VIM-4_ | Heavy metal resistance |
| Tn*6533*^202^ | Conjugative transposon | 109.0 | AP014651.1 | *bla*_IMP-1_ | Heavy metal resistance |
| Tn*6534*^202^ | Conjugative transposon | 118.7 | KX196168.1 | *bla*_GES-24_ | Heavy metal resistance |

*The transposon Tn*6286* was first identified in *Pseudomonas putida.*
